# Aligning a Household-Level Service Array Through a Jurisdiction-Wide Child Maltreatment Prevention Effort: Protocol for a Geospatial and Counterfactual Modeling Study

**DOI:** 10.2196/71997

**Published:** 2025-12-30

**Authors:** Jamaal Green, Brian Glass, Jordan Purdy, Dyann Daley

**Affiliations:** 1 University of Pennsylvania, 127 Meyerson Hall, 210 S. 34th Street, Philadelphia, PA, 19104, United States, 1 3018013109; 2 Counterbalance AI, Tualatin, OR, United States; 3 Predict-Align-Prevent, Grapevine, TX, United States

**Keywords:** counterfactual modeling, geospatial modeling, child maltreatment, public service provision, human services

## Abstract

**Background:**

Child maltreatment is associated with multiple negative outcomes at the individual and societal levels. Children experiencing maltreatment are at greater risk of a host of negative outcomes (eg, psychological disorders, substance use, violent delinquency, suicidality, and adverse educational outcomes).

**Objective:**

This study aims to prevent and ameliorate child maltreatment by using a combination of geospatial smoothing via a risk terrain modeling (RTM) framework and counterfactual modeling to identify risky areas and determine the optimal (re)allocation of services to maximally improve maltreatment outcomes.

**Methods:**

A 3-stage process is proposed that can iteratively be applied within a collaborating jurisdiction to enable responsive and sustained achievement of identified child welfare outcomes. This process makes use of 2 analytic approaches: geospatial smoothing via an RTM framework and counterfactual modeling. RTM is a spatial analytic approach that uses spatial machine learning methods to estimate the risk of maltreatment based on previous cases of maltreatment and risk factors of the built environment provided by the participating jurisdiction. Using previously validated cases of maltreatment as our target variable (eg, substantiated claims of abuse and neglect) and violent crime data and built environment data as our primary predictor variables, we estimate a series of machine learning models to geospatially smooth the historically identified places at increased risk of child maltreatment. Areas identified as higher risk receive extensive services associated with preventing or limiting child maltreatment, such as prenatal or postnatal care, subsidized daycare, and parental counseling. We make use of counterfactual explanation modeling to optimally align service allocation to maximally improve maltreatment outcomes for future service allocations within a collaborating jurisdiction. The technique leverages a statistical model associating household-level information with maltreatment outcomes to explore combinations of services that would be predicted to achieve optimal and practical recommendations for future service allocation efforts. Constraints can be introduced to this logic, such as service availability and cost. Algorithmic fairness is also a potential consideration during aggregation, with possibilities for both measuring and balancing metrics such as “recourse fairness.”

**Results:**

As of September 2025, a participating jurisdiction is being recruited.

**Conclusions:**

This protocol sets forth a novel approach for the allocation of supportive services for families at risk of child maltreatment through geospatial smoothing via an RTM framework and the maximization of service impact through a counterfactual explanation model. Child maltreatment is an unfortunate and ubiquitous issue in the United States. This proposal builds on jurisdiction-wide public health strategies to allocate services in a data-informed fashion and further align future iterations of the allocation strategy using outcomes-based counterfactual modeling at the household level. The flexibility of the proposed methodology enables its application regardless of the collaborating jurisdiction’s preferences and constraints.

## Introduction

### Background

In 2022, the United States Children’s Bureau reported 558,899 cases of child maltreatment and 1990 fatalities [1]. Although the rates of child sexual and physical abuse have declined in the United States since 1990 [2], the incidence of child maltreatment fatalities has risen since 2008. Child maltreatment is underreported [3], and actual fatalities are estimated to be 2 to 3 times higher than reported due to inconsistencies in definitions and reporting standards across states [4]. Child maltreatment is linked to the development of medical illness and psychiatric disorders, as well as poor education, employment, economic, interpersonal, and community outcomes [5-13].

The estimated cost per survivor of nonfatal child maltreatment is US $830,928 (2015), and the average lifetime cost associated with each child maltreatment fatality is estimated at US $16.6 million (2015). In 2015, the economic burden of child maltreatment in the United States was estimated at US $428 billion for substantiated cases and US $2 trillion for annually investigated incidents [14]. This significant economic burden becomes even more substantial when considered in the broader context of its impact on children, families, and communities.

Given the immense personal and societal costs of child maltreatment, it is important to design programs and policies to not only alleviate but also prevent maltreatment. Young children (ages 0‐3 years) are particularly exposed to the risks of maltreatment [15], and many children who die due to maltreatment are not known to child protection agencies [16,17]. Given these sobering facts, identifying places and families at increased risk of maltreatment may allow authorities to proactively intervene. Policies such as pre- and postnatal counseling, subsidized childcare, and parental counseling may all have protective effects with respect to potential maltreatment if agencies can successfully identify families at risk [18].

Beyond the general system-wide challenges in child welfare, maltreatment outcomes and preventive service availability demonstrate severe geographical inequities [19]. These service inequities span supportive and preventative services, including child welfare involvement itself [20]. Spatial modeling approaches can identify underserved areas and areas with disproportionately poor outcomes [21].

Therefore, this protocol aims to address three enduring issues: (1) targeting of primary prevention resources, (2) expansion of prevention programs to span risk factors, and (3) identification of the bundle of programs that effectively prevent child maltreatment for a given region or household. To accomplish this, the proposed study protocol seeks to identify areas with children at increased risk of experiencing maltreatment and to target these areas with extensive support programs to reduce and prevent maltreatment.

The study is composed of 3 stages, which can be iteratively applied within a collaborating jurisdiction to enable responsive and sustained achievement of the aforementioned goal. Stage 1 is the development of a smoothed risk surface of child maltreatment using spatial machine learning approaches within a “risk terrain modeling” (RTM) framework [22]. This stage also includes working with the collaborating jurisdiction to define child maltreatment, which can vary greatly depending on policy requirements, societal norms, and data availability [23-25]. Stage 2 comprises the resulting service allocation process for areas of increased estimated risk identified in Stage 1. Stage 3 uses a counterfactual explanation model (CEM) to optimally align service allocation to maximally improve maltreatment outcomes.

We propose the use of spatial risk modeling to improve targeting of primary prevention resources, coordination of the delivery of voluntary prevention programs that span individual and contextual risks using targeted universalism, and identification of the most cost-effective bundle of primary prevention programs using counterfactual modeling. It is important to note that this work is not concerned with simply validating whether individual support programs or their various combinations are effective in a general sense across a collaborating jurisdiction. This work seeks to expand the utility of classic program evaluation models by identifying household-specific combinations of available support programs that optimally improve household-specific outcomes.

### General Study Design

**Figure 1.**
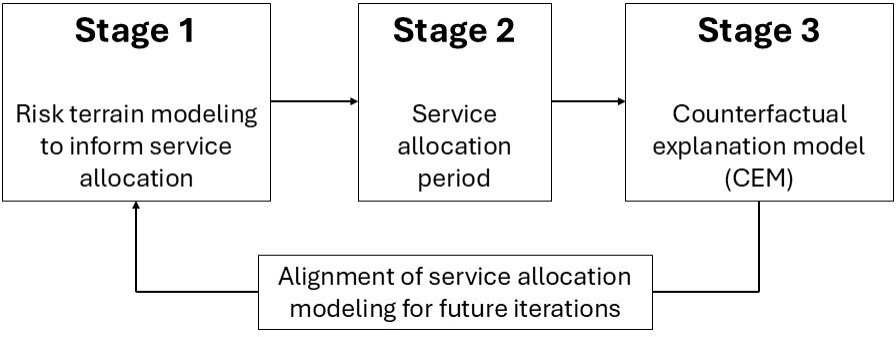
The 3 stages of the proposed study design.

The study protocol is divided into three stages: (1) Stage 1: geospatial service recommendations, (2) Stage 2: execution of the service array in the collaborating jurisdiction, and Stage 3: counterfactual modeling of service array impact (refer to Figure 1). The results from Stage 3 can then be used iteratively to inform the geospatial service recommendations moving forward. In Stage 1, geospatial information about historical child maltreatment events is leveraged in an RTM framework to produce a smoothed outcome surface of child maltreatment. The top areas of risk identified by the RTM (eg, the top quartile of “neighborhoods”) provide a recommended set of locations to receive expanded child welfare–related services. In Stage 2, the services are offered and allocated within the collaborating jurisdiction guided by the geospatial service recommendation map from Stage 1. After a period of service delivery of sufficient duration to allow for a long-arc child maltreatment outcome window (eg, 5 years total, with 3 years of service delivery and a 2-year outcome window), Stage 3 involves the development of a CEM to develop actionable jurisdiction-wide recommendations based on the impact of the service array on child maltreatment in the jurisdiction. Rather than provide a simplified pre-post evaluation of service effectiveness, CEM offers a tailored approach to identify optimal service arrays at the household level, thus further aligning the geospatial service recommendations offered in Stage 1. Several details of the protocol, including the specific services offered, duration of Stage 2, and the final format of the actionable recommendations, will be determined during the design phase, which will involve feedback and specifications provided by the collaborating jurisdiction. This study protocol seeks to propose a methodological framework that is robust to the various configurations and challenges that may arise during the final determination of the study details, which will be highly dependent on the governing body in the collaborating jurisdiction.

### RTM: Theoretical Background and Use Cases

RTM is a spatial analytic approach developed by Caplan [26] for the purpose of estimating spatial risk with respect to environmental determinants of crime. While RTM has roots in criminology literature, the basic approach is an application of existing spatial analytic approaches. The ultimate goal is the identification of risky places as opposed to individuals. The environmental criminology literature has long argued that certain environmental features can increase or decrease the risk of certain crimes occurring. Brantingham and Brantingham [27] theorized that there are 2 major types of features in the built environment that influence crime: crime attractors and crime generators. Crime generators are areas that attract numerous people and thus offer increased opportunities for crime. Stadiums or entertainment districts are 2 examples of crime-generating spaces. Crime attractors, conversely, are places that offer opportunities for crime and are attractive to potential offenders because of the opportunity to engage in certain types of crime. Open-air drug markets are an example of such a place. RTM, then, is an application of the basic logic set forth by Brantingham and Brantingham [27] through the use of spatial predictive modeling.

The classic use cases for RTM include estimating neighborhood risk profiles with respect to a variety of crime types. Violent crimes, such as shootings or robberies, are often studied [28]. Recently, RTM has also been used to identify places at higher risk of child maltreatment [22,29].

### Service Allocation Period

The service allocation period will rely on the geospatial analysis from Stage 1 and involves the actual service delivery of various prevention programs to households in the jurisdiction. The jurisdiction-wide service delivery expansion effort is influenced by previous work in public health strategies for the deployment of large-scale service delivery efforts. Refer to the “Stage 2-Service Allocation” section for further details.

### CEMs: Theoretical Background and Use Case Examples

CEMs are statistical models of real-world phenomena that can produce actionable recommendations to maximize or minimize the probability that some alternative outcome would have occurred. CEMs have generated considerable recent interest due to (1) their ability to resolve interpretations from “black box” models [30,31], (2) their structural alignment with both scientific and everyday reasoning about the world [32], and (3) their ability to offer deployable and testable courses of action for policymakers and practitioners. CEM methodologies can produce counterfactual courses of action for a single observational input (eg, how a loan applicant can improve their standing, how a medical course of action can improve the survival of an intensive care unit (ICU) patient, or how a given service combination can improve the child welfare outlook of a single household).

The classic use case example for CEMs is the financial loan scenario: consider a statistical model that predicts whether a lendee will repay a loan within a given timeframe. By perturbing the input factors in the statistical model, the lending institution can discover a set of “solutions” that would be predicted to convert an unsuccessful loan applicant into a successful loan applicant, thus offering recommendations to rejected applicants. Another recent example from the field of health care is the proposal for CEM in an intensive care unit [33]. In the proposal, a CEM built on historical data offers treatment recommendations to maximize patient survivability. If a patient is predicted to have low chances of survival upon ICU intake, the CEM offers a treatment array sequence to maximally improve the chance of survival. Thus, researchers leveraged a statistical model linking treatment combinations to health outcomes to provide actionable individual-level treatment array recommendations.

### Counterfactual Analysis in Child Welfare

Statistical approaches to investigate the decision-making in the area of child welfare often deploy methodology that uses the term “counterfactual.” One such approach is counterfactual causal modeling, which aims to determine hypothetical outcomes under different child welfare decision policies [34,35]. Another approach uses an economic formulation of “counterfactual” analysis to investigate alternative hypothetical mechanisms for assigning child welfare cases to government workers [36]. “Counterfactual” modeling has also been used for evaluating the effectiveness of a substance use intervention program by generating simulated treatment and control groups [37]. While these approaches are counterfactual in the general sense, they differ from the approach specified in this work. Specifically, this work seeks to model service delivery information and outcomes to optimize service array combinations at the family level. It is possible that jurisdictions have internally planned or developed similar methodology, but the authors are unaware of any publicly acknowledged efforts to develop or deploy such a technique.

### Characteristics of CEMs

A variety of theoretical work has considered the evaluation of a CEM’s quality [38]. This previous work generally considers 4 domains for the evaluation of a given CEM: validity, proximity, diversity, and actionability [39].

Validity can refer both to the performance (eg, area under the curve, accuracy, and smoothness of the data manifold) of a CEM’s underlying statistical model, as well as the CEM’s ability to provide truly counterfactual predictions [39]. Individual predictions are truly counterfactual when the resulting outcome is in a different class than the original outcome (ie, perturbing the features of a given input vector results in a different predicted binary outcome). Thus, statistical models whose predicted outcomes are invariant to alterations in the input vector values are not valid for use as a CEM.

Proximity refers to the minimally required alteration of an input vector CEM to generate effective counterfactuals (ie, altered input vectors which result in a different prediction class). CEM models with superior proximity generate counterfactual input vectors using minimal perturbations to the original input vector [40-42].

Diversity refers to the size or cardinality of the set of counterfactual options resolved by a CEM [42]. For example, for a given individual input vector (ie, the quantitative description of a household’s features and services), a CEM might recommend a large and varied set of counterfactual feature configurations that all successfully convert the prediction to the more ideal class. While not necessarily a detriment, large sets of “other possible worlds” offer a conundrum to CEM deployment, as many differing and often competing courses of action must be further compared and evaluated. This challenge, known as the Rashomon Effect [43], requires practitioners to select a single available counterfactual option from a large and varied set of options. The development of a framework to compare these options is available in the “Compute Counterfactual Service Arrays” section.

Actionability refers to the level of controllability of the input vector features perturbed by the CEM [44]. For example, an ICU treatment model that counterfactually recommends that a patient become taller is not actionable. This proposal seeks to maximize actionability by only perturbing input vector features that relate to the application of specific family services.

## Methods

### Overview

The study consists of 3 stages, which can be iteratively applied within a collaborating jurisdiction. Stage 1 is the development of a smoothed risk surface of child maltreatment. Stage 2 is the resulting service allocation process for areas of heightened risk identified in Stage 1. Finally, Stage 3 uses a CEM to determine optimally aligned service allocation for future iterations of the service array.

### Stage 1-RTM

#### Overview

The objective of Stage 1 is neither to predict when or where the outcome will occur nor to identify regions of latent risk, but rather to apply RTM to initially identify the regions that might reasonably be expected to benefit from service allocation. To this end, we propose smoothing the historical outcome surface across the jurisdiction via the development of a geospatial machine learning algorithm, where regions corresponding to higher values from the trained smoother will inform the initial service allocation encompassed by Stage 2.

Within the jurisdiction of interest, the observational units of this geospatial smoother are regular polygons, the size of which will depend upon the collaborating jurisdiction. Henceforth, these observational units are referred to as “fishnet” grid cells. This fishnet is a regular grid of polygons (in this case, squares) with an area sufficient for block-to-neighborhood-level modeling but smaller than standard census geographies. The outcome being smoothed will pertain to child maltreatment, as defined by the collaborating jurisdiction, with the specific form (eg, rate, count, and so on) likely being determined after initial exploratory data analysis. The feature set will consist of variables based on the built environment, crime, and census data both within the cell and within neighboring cells, as well as the value of the outcome in neighboring cells (ie, auto-features), where a variety of isotropic neighborhood structures will be considered (eg, first-order, second-order, and so on).

RTM requires appropriately robust models that describe the environment. Traditionally, this has involved the use of administrative data sources, such as US census data, arrest or crime data from local law enforcement, as well as “environmental” data. For this study, our primary datasets will include address-level child welfare data on substantiated cases of maltreatment (eg, neglect, physical, sexual abuse, and so on), local law enforcement arrest data for violent crimes (eg, assaults, domestic disturbance calls, homicides, and so on), socioeconomic data from the US Census, and built environment data from the jurisdiction’s open data sources as well as state-level administrative data where possible.

Local socioeconomic environmental variables from the US Census will be represented by the Neighborhood Deprivation Index [45]. This index is a validated and supported composite measure of neighborhood deprivation derived from the first principal component of a set of 20 census variables. The final index makes use of 8 of those variables found in the first component: share of males in management and professional occupations, share of crowded housing, share of households in poverty, share of female-headed households with dependents, share of households on public assistance, share of households earning less than US $30,000 a year, share of the population with less than a high school diploma, and share unemployed. Built environment data will include recognized risk factors in child maltreatment, including alcohol-serving establishments (both bars/restaurants and liquor stores), cannabis dispensaries (where recreational cannabis is legal), dangerous buildings from local code violations data, transit stops, and supportive/protective land uses such as community centers, daycares, and houses of worship.

Two approaches to creating the smoothed surface will be considered, with goodness-of-fit measures used to identify the better of the 2. The first, kernel density estimation [26], is expected to be inferior and will serve as the baseline approach. The second, a tree-boosted learner (eg, XGBoost [Extreme Gradient Boosting] [46]), is more complicated but is expected to be superior. For the boosted learner, k-fold cross-validation—with the spatial neighborhood structure dictating the partitioning of the folds—will be used. All risk factors will be included in the final model. Tree-based models such as XGBoost are generally robust to issues of multicollinearity and other risks of high-dimensional data. Hyperparameter tuning will be led by a grid search based on a range of hyperparameter values [47]. The combination of the grid search and spatial-based k-fold validation should allow for the estimation of a strong predictive model while guarding against overfitting and excessive residual spatial autocorrelation. For this protocol, the smoother produced through RTM is used to identify areas of heightened risk of child maltreatment to guide the initial round of service allocation decisions. Model predictive accuracy will be assessed using a spatial cross-validation approach.

#### Algorithmic Fairness of Geospatial Smoother

Within the paradigm of person-level algorithms and related decision-support tools, there exists a robust literature on algorithmic fairness. Among other things, this literature posits numerous definitions of algorithmic fairness [48,49] for an identified, and potentially multidimensional, protected attribute, along with a number of mitigation procedures for “correcting” the output of such person-level algorithms with respect to the protected attribute [50-52]. Unfortunately, within the paradigm of geospatial algorithms and related decision-support tools, there is a relative dearth of literature [53] pertaining to algorithmic fairness.

In light of this reality, one approach sometimes used is an assessment of the geospatial algorithm’s generalizability across coarse categorizations of poverty (high- vs low-poverty), race (majority White vs majority non-White), or some combination thereof, based on census data [54]. Such assessments are akin to a type of algorithmic fairness audit, but provide no clear course of action in the face of poor generalizability.

Here, we propose a relatively simple approach encompassing the algorithmic fairness of the geospatial smoother and the corresponding identification of fishnet cells for service allocation. Our goals with this procedure are to enable a simple, coarse auditing of the geospatial smoother’s outputs across a census-derived protected attribute and to provide a means for the subsequent and corresponding equitable allocation of services across the levels of the protected attribute.

The approach to algorithmic fairness consists of 2 steps. In Step 1, unsupervised machine learning (eg, clustering) is used to identify “levels” (eg, clusters) of a protected attribute. Such unsupervised learning would use relevant (as identified by the jurisdiction of interest) features from the census data (eg, racial percentages, poverty percentages, and so on). If the proposed unsupervised learning “fails” (ie, is uninformative for identifying groups or levels of a protected attribute), then we will default in Step 1 to a poverty- and/or race-based protected attribute consisting of only a few levels. In Step 2, the limited number of fishnet cells identified for allocation of services within Stage 2 is based on protected attribute-level stratification of the geospatial smoother’s output. Such identification of fishnet cells for Stage 2 is consistent with an equitable allocation of services, where the form of equity is ultimately determined by the collaborating jurisdiction. For example, a jurisdiction could elect to allocate each level of the protected attribute an equal number of fishnet cells receiving services, with the specific fishnet cells within each protected attribute level being identified according to higher values of the geospatial smoother.

While this proposed approach to algorithmic fairness related to the geospatial classifier is relatively “simple,” it is important to recognize that the norm is to ignore considerations of algorithmic fairness altogether. Furthermore, while there may understandably be concern that this geospatial smoother may inadvertently perpetuate existing biases, either with or without our proposed approach to algorithmic fairness, it is important to recognize that the proposed “intervention” (ie, Stage 2) consists of providing optional supportive services rather than imposing nonoptional punitive measures. Hence, the proposed approach elevates the standard by incorporating algorithmic fairness and inherently mitigates fairness concerns by allocating optional and supportive services.

### Stage 2-Service Allocation

The service array provided to the households within the fishnet cells identified in Stage 1 will be finalized with input from the collaborating jurisdiction, although the following list presents a core set of service programs and/or service types, with the target population included for each item. The service array will be selected to impact the various known major risk factors for child maltreatment or adverse childhood experiences. These services include nurse-family partnership for pregnant people and young families [55], cure violence (neighborhood) [56-58], crime prevention through environmental design (neighborhood) [59-61], universal basic income (household) [62,63], early head start (child and young families) [64,65], pregnancy prevention (eg, Upstream USA; adults and families) [66,67], mobile medical clinics (household) [68,69], crisis intervention teams (neighborhood and household) [70], free college tuition (both municipal or state) [71,72], and Stewards of Children: Darkness to Light Training (children) [73].

Each service has a demonstrated impact on one or more of these major risk factors. This comprehensive, multiservice, jurisdiction-wide approach uses a public health strategy [74,75] to expand preventive services to households that may not already be known to the collaborating jurisdiction’s child welfare system. The entire set of services chosen by the collaborating jurisdiction will be available to every qualified household within each fishnet cell identified in Stage 1.

Data records will track household-level service delivery and participation, allowing for the quantification of service array information for use in Stage 3. A centralized service allocation database will be maintained by the jurisdiction. To properly quantify service delivery in a manner that is quantifiable for the CEM, data must include the service delivery period and the type and/or subtype of the service delivered by the provider. In the feature specification phase of the CEM construction, the service delivery features will be identified as Boolean values (ie, did or did not receive the service). Therefore, it may be necessary to define a single service type as multiple mutually exclusive features. For example, if data investigation reveals a distribution of service delivery periods for a specific service type, further discussion with the jurisdiction and service provider may motivate the differentiation of a given service type into 2 or multiple features (eg, service type A [≤1 month], and service type A [>1 month]). Careful analysis of the service delivery tracking database, along with input from the collaborating jurisdiction and service providers, will be required for the appropriate specification of the service type features used in the CEM.

### Stage 3-Service Alignment Recommendation via CEM

#### Maltreatment Classifier Model Construction and Selection

The first step of Stage 3 is to develop a statistical model to associate available and quantifiable features with the selected child welfare outcome of interest. Model specification and selection will consider the 4 characteristics of CEMs identified above. While data availability details will not be finalized until the completion of Stages 1 and 2 in the collaborating jurisdiction, previous work suggests it is reasonable to anticipate that machine learning models can be trained to associate quantifiable household factors with child maltreatment outcomes with high performance, high algorithmic fairness, and high computational efficiency [52,76,77]. One novel contribution of this study protocol will be to extend the use of such models to inform the alignment of jurisdiction-wide service deployment strategies by maximizing the potential for positive outcomes at the household level.

#### Observational Unit and Data Universe

The granularity of the statistical model will be the household level. All households in the collaborating jurisdiction with at least one child member will comprise the data universe. The data set used to train and validate the statistical model will consist of the union of three mutually inclusive subsets of the data universe: Set 1: Households eligible to receive services as identified in Stage 1 of the protocol, Set 2: households that actually received services in Stage 2 of the protocol (some of which may not have been in Set 1), and Set 3: households found in the Statewide Automated Child Welfare Information System (SACWIS) [78]. Thus, the union of these 3 sets encompasses all households who either were eligible to receive services and/or are known to have the necessary SACWIS records to define an outcome. Households in the jurisdiction universe that fall out of this union set will be available as a counterfactual inference set. That is, despite not having a defined service record, their features will be available to construct an input vector for the statistical model, allowing for a predicted outcome and, in turn, an optimized counterfactual service array.

#### Sources of Model Features

A feature engineering stage will attach available quantifiable information to each household observation. These features will reflect information drawn from four general sources: Source (1) Household-level data available from consumer and market data, Source (2) local built environment data attached to nearby households, Source (3) Governmental reporting and services data, and Source (4) the household’s actual service array experience during Stage 2. During counterfactual optimization, Sources 1 and 2 remain static. At the same time, Source 4 (the service array) is perturbed in order to minimize (or maximize) the predicted probability of the chosen child welfare outcome of interest. In this way, maximum actionability is preserved, since no household-level features beyond the controllable service array are perturbed as part of the counterfactual modeling. Importantly, administrative data from the SACWIS system will not be included as model features. This allows for the counterfactual model to be deployable for households that have not been involved with the collaborating jurisdiction’s child welfare system. Governmental reporting and services data (Source 4) can act as important predictors as well as inform a potential post hoc outcomes analysis (refer to “Stage 2-Service Allocation”). The following list presents potential data elements for Source 4: (1) health care/hospital may include indicators such as preterm birth, very low birth weight, failure to thrive, neonatal abstinence syndrome, pediatric physical/developmental disabilities, teen birth, preventable hospital admissions, Prevention Quality Indicators (hospital visits), Pediatric Quality Indicators (area level), psychiatric hospitalizations, injuries from violence, pediatric lead poisoning, maternal morbidity/mortality, substance abuse, substance-related overdoses, accidental deaths, sexual violence/rape, and prenatal care visits; (2) data from the Department of Health/Medical Examiner may encompass infant/child deaths, premature deaths, excess mortality, and marriage/divorce; (3) crime/emergency service records may include arrests, incarcerations, domestic disturbance/violence, assault, gunshot/shooting/stabbings, intoxication, drug abuse/manufacturing, animal abuse/control, sexual assault/rape, arson, DUI, homicide, harassment, juvenile kidnapping, reckless driving, suicide, and welfare checks; and (4) education/employment data may capture graduation rates, truancy rates, higher education/trade school matriculation, kindergarten readiness, third-grade reading level, unemployment, and poverty indices.

#### Model Outcome

The outcome will be defined via a joint venture with the collaborating jurisdiction’s governing body and will relate to child maltreatment at the household level. Defining child maltreatment is a classic family-resemblance challenge, with no universally agreed-upon definition having emerged from over a century of academic and governmental interest in the phenomenon [25]. Previous work has considered various maltreatment-related outcomes that vary by intensity and prevalence, such as reports or referrals of child maltreatment (ie, community members reporting alleged child abuse or neglect to a child welfare agency), whether a child protective services investigation occurred for an alleged child survivor in the household, whether a child protective service investigation determined substantiated maltreatment in the home, and whether a child in the home was removed and placed into some form of substitute care as a ward of the state [23,24]. Outcome windows can also vary in length, although typical durations include 6 months, 1 year, and 2 years [52,79-81]. Outcome availability will differ between households depending on whether the question at hand is a question of reporting potential maltreatment, whether an investigation should occur, or the question of substantiation of child maltreatment 3, although it will be possible to both predict and define an outcome for each observation. For example, a household that was eligible for services and received services but was not found in the SACWIS system (ie, a member of Sets 1 and 2, but not Set 3) will be defined as “maltreatment absent,” that is, there was no detected household maltreatment. However, it will still be possible to calculate a predicted probability of “maltreatment present,” thus allowing for defining a counterfactual service array that could have further decreased any potential for maltreatment present in the household.

#### Model Selection Criteria

Best practice will be followed in order to construct and select between statistical model specifications that best satisfy the requirements for an acceptable associative model for use in the CEM stage [82-84]. This best practice includes (1) exploring a range of diverse machine-learning classifier types (eg, XGboost, support vector machines, neural networks, and so on), (2) comparing classifier performance on a validation set (eg, area under the curve, accuracy, specificity, sensitivity, and so on), (3) comparing the performance-fairness tradeoff for each model, with the aim of achieving large gains in algorithmic fairness at minimal cost to performance, (4) assessing computational demands (as the CEM stage would ideally use an exhaustive search over thousands of perturbed input vectors for each observation), and (5) ensuring the ability of the statistical model to underlie a CEM that produces counterfactual results that are valid, proximal, diverse, and actionable (refer to “Characteristics of Counterfactual Explanation Models” section above).

#### Potential Algorithmic Fairness Correction

The resulting statistical model will produce predicted probabilities that will be used ordinally within an observation’s counterfactual array (refer to the “Compute Counterfactual Service Arrays” section). However, it is possible that the final deployed CEM will require between-observation comparison at the aggregation stage to meet the use-case requirements selected in collaboration with the jurisdiction. In the case that predicted probabilities will be directly compared between observations, and therefore between households at different levels of a protected attribute, algorithmic fairness corrections will be considered. The construction of such correctional procedures will follow the guidance of the well-established literature referenced in the “Algorithmic Fairness of Geospatial Smoother” section above [52]. In particular, the collaborating jurisdiction will determine, ideally with input from corresponding stakeholders, both the protected attribute of interest and the relevant definition of algorithmic fairness. Once these decisions are made, one or more appropriate correction procedures from the peer-reviewed literature can be identified and implemented to improve the fairness of the service-allocation recommender across the levels of the protected attribute.

### Compute Counterfactual Service Arrays

The statistical model identified above through the selection criteria will become the underlying classification mechanism of the CEM. This CEM will produce service alignment recommendations by computing counterfactual service arrays for each historical observation at the household level. This will be accomplished by optimizing the service array vector for each household observation with respect to certain criteria or constraints, such as minimizing the predicted probability of the maltreatment outcome while also minimizing the number of additional services required. The following computational methodology outlines how this is accomplished: (1) an input vector is constructed for each household observation, which includes both the household characteristic features and the binary service array features (ie, a 0 signifies the household received Service A, while a 1 signifies the household did not receive Service A); (2) the permutations of the binary service array vector are considered to create a set of (at most) 2^n^ candidate input vectors (where n is the number of available services), one of which represents the actually received service array; (3) a predicted probability of the maltreatment outcome is computed for each candidate input vector; and (4) each of the 2^n^ candidate input vectors is quantified in terms of CEM characteristics for use in the aggregation stage. Ideally, a brute-force search will be used in this protocol to exhaustively consider all service array permutations, precluding various challenges with CEM deployment associated with the possibility of missing globally optimal regions of the search space [85]. However, if computational demands outweigh the available resources, nonexhaustive optimization algorithms (eg, mixed-integer programming [86] or genetic algorithm search [87]) are available to identify counterfactual service arrays that significantly lower the inferred probability of maltreatment.

In Step 4 above, each candidate input vector (of the 2*^n^* defined for each household observation) is described by certain characteristics. These characteristics are (1) the change in predicted probability of the maltreatment outcome between original input vector and the candidate input vector (eg, +4% and −10%), (2) the proximity of the candidate service array vector to the original service array vector in Hamming distance (ie, the number of services that differ between the original and the candidate counterfactual under consideration [88]), and (3) the proximity of the candidate input vector to a known alternative input vector from the newly predicted class, representing a proxy for generalizability confidence (by Gower’s distance [89], with 1 representing equality and 0 representing maximal distance in the set). In this way, the full set of counterfactual service array candidates for a given household observation can be sorted, filtered, and evaluated to serve specific purposes in the Recommendation Aggregation stage.

For example, consider a household observation whose maltreatment outcome was positive (with retroactive predicted probability of 85%) and whose actual service array vector was {0, 0, 1, 0, 1, 0} (ie, in Stage 2, the household received services C and E). For clarity, consider 3 members of its resulting 2*^n^* candidate input vectors. Table 1 describes these candidate input vectors and the resulting CEM characteristics A-D for each candidate. Identifying a single recommendation from the resulting counterfactual candidates requires developing certain standards or rules. These criteria will be developed in conjunction with the collaborating jurisdiction to maximally adhere to their pragmatic considerations, such as resource constraints. For this example, consider how these candidates might be compared to one another. Candidate 1 reduces the predicted probability by 5%, which is less than the other 2 candidates; however, it requires only 1 additional service and is also very similar to a known observation in which maltreatment was actually prevented. Candidate 2 reduces the predicted probability by 35%, requires a net change of 1 service (1 subtracted and 2 additional), and has moderate similarity to a known observation in the alternative class. Candidate 3 reduces the predicted probability by 40%, more than the other candidates, but requires 3 additional services and is minimally similar to a known observation in the alternative class. If the determined considerations called for identifying recommendations that would require minimal additional services and maximize proximity to known observations of the maltreatment-prevention class, then Candidate 1 would be selected. If the determined considerations called for identifying recommendations that would maximize the reduction of predicted maltreatment, regardless of the number of additional services and regardless of the confidence provided by proximity to known observations, then Candidate 3 would be selected. A balanced approach might result in the selection of Candidate 2. In this way, the comprehensive quantification of each candidate result works to anticipate the myriad potential weighting scenarios from which one will be chosen as the guiding principle for selecting household-level counterfactual recommendations.

**Table 1.** Example comparison of 3 counterfactual candidates computed from an observation using the counterfactual explanation model (CEM). A walkthrough of this table is available in the “Compute Counterfactual Service Arrays” section.

Input vector description	Service array vector	Predicted probability of maltreatment	Change in predicted probability of maltreatment	Change in services required	Proximity to known member of the No-Maltr. class
Actual	{0,0,1,0,1,0}	85%	—	—	0.31
CEM^a^ candidate 1	{0,0,1,1,1,0}	80%	−5%	Net 1 (+1)	0.99
CEM candidate 2	{1,1,0,1,1,0}	50%	−35%	Net 1 (+1,‐2)	0.67
CEM candidate 3	{1,1,1,0,1,1}	45%	−40%	Net 3 (+3)	0.42

a CEM: counterfactual explanation model.

### Technical Considerations

Technical challenges may arise, such as entity resolution and data quality issues. Because joining the disparate datasets is critical, the challenge of entity resolution may require the deployment of one or more technical solutions. These solutions can range from fuzzy matching to deep learning [90,91]. In addition, data quality issues such as missingness, time period availability, and confounds due to policy change over time will likely be present. Expertise and experience in the data engineering of administrative data will be vital to ensure maximal validity and reliability of the results [92,93].

### Change Management Considerations

Access to the family-level recommendation information provided by this protocol will undoubtedly constitute an implementation challenge for the jurisdiction. Thus, it will be critical to engage with the jurisdiction’s change management entities, including implementation teams, continuous quality improvement teams, and communication teams, to develop a plan to execute the resulting recommendations. Conversations with these teams will expose the practical, policy, and legal challenges that must be addressed by the business framework surrounding the use of the information [94,95].

### Ethical Considerations

The execution of this protocol will require input from the collaborating jurisdiction regarding data governance and ethical considerations. While requirements for ethics review approvals and informed consent differ greatly for governmental agencies relative to public research institutes, the protocol must comply with the jurisdiction’s data governance requirements. This includes data security, data use agreements, data privacy, and the masking of personally identifiable information.

A central ethical concern in the area of predictive analytics and child welfare is the stigmatization of individuals, households, and neighborhoods via automated labels such as “high risk” [96]. To minimize stigmatization, the use of technical and statistical terminology should be separated from the communication and design concepts that govern any public-facing or user-facing material, dashboards, or deployable implementation that results from this protocol [97,98]. For example, one practical deliverable from this protocol could be a family-specific report that can guide a human services caseworker or supervisor to additional services that might promote more positive outcomes for a family. Framed as a guide for improving the family’s outcomes, the material should avoid terminology such as “high risk.” In summary, the protocol must adhere to the specific data governance and ethical considerations of the collaborating jurisdiction.

## Results

As of September 2025, a participating jurisdiction is under recruitment.

## Discussion

### Principal Findings

The household-level counterfactual recommendations described in the previous section will be aggregated into actionable recommendations. The primary actionable recommendation will directly inform future iterations of Stage 1 by providing a model-informed approach for determining eligible regions. Other secondary recommendations are also possible if the final project scope calls for other ways to aggregate the results to fit various project goals. The overall conceptual method for generating these recommendations is to aggregate household-level counterfactual service array candidates into an actionable result [99].

There are several methods and strategies for aggregating the resulting household-level counterfactual service arrays. The first step is to select, for each household observation, which of the 2*^n^* service array permutations should be selected. The previous section details how this selection logic can be specified to reflect the jurisdiction’s overall project goals and constraints. The second step is to aggregate or “roll up” the household-level counterfactual service arrays into spatial subregions. These subregions could be the fishnet grids specified in Stage 1, or any other chosen subregion definition, such as ZIP code, census tract, or school district. Bespoke logic can determine the most pragmatic approach for grouping service recommendations into subregions. The simplest method is to, for each service type, sum the number of households in the subregion that were determined to benefit from the service. Constraints can be introduced to this logic, such as service availability and cost. Algorithmic fairness is also a potential consideration during aggregation, with possibilities for both measuring and balancing metrics such as “recourse fairness” [100]. Finally, these results are organized into an actionable recommendation strategy that can be provided to the jurisdiction to improve its future service delivery.

### Comparison With Previous Work

This protocol sets forth a novel approach for the allocation of supportive services for families at risk of child maltreatment through geospatial smoothing via an RTM framework and the maximization of service impact through CEM. Child maltreatment is an unfortunate and ubiquitous issue in the United States. This proposal builds on jurisdiction-wide public health strategies to allocate services in a data-informed fashion and further align future iterations of the allocation strategy using outcomes-based counterfactual modeling at the household level. The flexibility of the proposed methodology enables its application regardless of the collaborating jurisdiction’s preferences and constraints.

### Strengths and Limitations

While there are challenges in setting up and implementing the proposed 3-stage process, including, but not limited to, data collection, privacy, and stigmatization, the potential benefits within a collaborating jurisdiction are numerous. Achievable benefits include, among other things, reduced child welfare involvement and reduced adverse childhood experience scores, more focused and efficient allocation of services and programs, as well as increased school attendance and graduation rates within the neighborhoods of the collaborating jurisdiction.

It is worth noting that while the objective of the presented protocol is to reduce child maltreatment within the collaborating jurisdiction, post hoc descriptive analyses related to changes in jurisdiction-wide outcomes known to be associated with child maltreatment (eg, adverse childhood experience scores, violent crime, and so on; refer to “Sources of Model Features” section for an expanded list) are possible and may be of interest to both the collaborating jurisdiction and corresponding service providers. For example, the Cure Violence program will likely be interested in whether aggregate measures of violent crime in the collaborating jurisdiction have declined pre- versus post implementation of a given iteration of the implemented service array. Importantly, however, whether such associated outcomes are causally improved as a result of the implemented service array cannot be nor is intended to be answered by the presented protocol. Such associated outcomes may or may not be causally related to child maltreatment, and therefore, reducing child maltreatment may not result in, for example, a reduction in violent crime across the collaborating jurisdiction. Regardless, such post hoc analyses are possible and can be informative for both the collaborating jurisdiction and corresponding service providers.

### Future Directions

This proposal represents a maturation of machine learning and artificial intelligence in the public policy space. By leveraging the historical information in a narrowly focused machine learning approach, jurisdictions can develop data-driven recommendation tools that continuously improve over time. As explained in the “Strengths and Limitations” section, future stages of this approach can involve an enhanced underlying machine learning model based on new incoming data and outcomes from the previous stages. In this way, the general approach represents a continually evolving strategy rather than a single piece of analysis.

## Abbreviations

CEM: counterfactual explanation model
ICU: intensive care unit
RTM: risk terrain modeling
SACWIS: Statewide Automated Child Welfare Information System
XGBoost: Extreme Gradient Boosting
